# The human cytomegalovirus glycoprotein pUL11 acts via CD45 to induce T cell IL-10 secretion

**DOI:** 10.1371/journal.ppat.1006454

**Published:** 2017-06-19

**Authors:** Jasmin Zischke, Panagiota Mamareli, Claudia Pokoyski, Ildar Gabaev, Sabine Buyny, Roland Jacobs, Christine S. Falk, Matthias Lochner, Tim Sparwasser, Thomas F. Schulz, Penelope C. Kay-Fedorov

**Affiliations:** 1 Institute of Virology, Hannover Medical School, Hannover, Germany; 2 German Center for Infection Research (DZIF, TTU-IICH), Hannover-Braunschweig Site, Hannover, Germany; 3 Institute of Infection Immunology, TWINCORE, Centre for Experimental and Clinical Infection Research; a joint venture between the Medical School Hannover (MHH) and the Helmholtz Centre for Infection Research (HZI), Hannover, Germany; 4 Department of General, Visceral and Transplantation Surgery, Hannover Medical School, Hannover, Germany; 5 Cambridge Institute for Medical Research, University of Cambridge, Cambridge, United Kingdom; 6 Department of Clinical Immunology and Rheumatology, Hannover Medical School, Hannover, Germany; 7 Institute of Transplant Immunology, IFB-Tx, Hannover Medical School, Hannover, Germany; Blumburg Institute, UNITED STATES

## Abstract

Human Cytomegalovirus (HCMV) is a widespread pathogen, infection with which can cause severe disease for immunocompromised individuals. The complex changes wrought on the host’s immune system during both productive and latent HCMV infection are well known. Infected cells are masked and manipulated and uninfected immune cells are also affected; peripheral blood mononuclear cell (PBMC) proliferation is reduced and cytokine profiles altered. Levels increase of the anti-inflammatory cytokine IL-10, which may be important for the establishment of HCMV infections and is required for the development of high viral titres by murine cytomegalovirus. The mechanisms by which HCMV affects T cell IL-10 secretion are not understood. We show here that treatment of PBMC with purified pUL11 induces IL-10 producing T cells as a result of pUL11 binding to the CD45 phosphatase on T cells. IL-10 production induced by HCMV infection is also in part mediated by pUL11. Supernatants from pUL11 treated cells have anti-inflammatory effects on untreated PBMC. Considering the mechanism, CD45 can be a positive or negative regulator of TCR signalling, depending on its expression level, and we show that pUL11 also has concentration dependent activating or inhibitory effects on T cell proliferation and on the kinase function of the CD45 substrate Lck. pUL11 is therefore the first example of a viral protein that can target CD45 to induce T cells with anti-inflammatory properties. It is also the first HCMV protein shown to induce T cell IL-10 secretion. Understanding the mechanisms by which pUL11-induced changes in signal strength influence T cell development and function may provide the basis for the development of novel antiviral treatments and therapies against immune pathologies.

## Author summary

Human cytomegalovirus (HCMV) infects from 45% to 100% of people worldwide, depending on local socio-economic factors. Although usually harmless in healthy individuals, infection with HCMV can cause severe disease in people with weakened or immature immune systems such as transplant recipients and newborns. The establishment and maintenance of life-long infections by HCMV are greatly aided by its ability to modulate the host’s immune system during both active and latent infection; infected cells are masked and both infected and uninfected immune cells have their functions manipulated. One effect of HCMV infection is the induction of the cytokine IL-10, a secreted protein that suppresses many antiviral responses. Here, we identify a viral protein, pUL11, which can induce IL-10 expression by T cells and reduce the production of mediators of inflammation. pUL11 interacts with CD45, an immune system regulator that controls the sensitivity of T cells and has been linked to IL-10 production. We show that pUL11 can likewise affect T cell responses to stimuli, depending on its concentration, and suggest that this underlies its functions. pUL11 is the first viral protein known with this mechanism and further understanding of its effects may lead to the development of novel antiviral therapies and also help in the treatment of immune system disorders.

## Introduction

Human Cytomegalovirus (HCMV) is a ubiquitous human pathogen with a high seroprevalence of between 45 and 100% worldwide [1]. While mostly asymptomatic in healthy individuals, infection with HCMV in immunocompromised individuals can cause severe disease or death. Congenital HCMV infection, for example, results in permanent disability in an estimated number of approximately 8,000 children per year in the US and 1,100 in France [2]. HCMV primary infection or reactivation from latency is also a major problem for both stem cell and solid organ transplant recipients, as it can cause clinical disease and also have indirect effects on survival, including increasing the likelihood of the occurrence of secondary bacterial, fungal and viral infections due to CMV-mediated myelosuppression [3–5].

The intricate and complex changes wrought on its host’s immune system during all stages of HCMV infection are well documented [6]. Infected cells are masked from recognition and have their functions manipulated to the benefit of the virus. Uninfected immune cells are also affected; a generalized myelosuppression has been described, the ability of peripheral blood mononuclear cells (PBMC) to proliferate in response to stimuli is reduced and the cytokine profile is altered [5,7–9]. The mechanisms by which uninfected cells are manipulated are not yet fully understood but appear to involve both direct contact with infected cells and also the actions of secreted factors [10,11]. Levels of the anti-inflammatory cytokine IL-10 increase during both productive and latent stages of infection, which may be important for the establishment and maintenance of a stable HCMV infection. IL-10 levels are positively associated with the incidence and duration of viraemia in HCMV positive transplant recipients [8,9,12–15]. Interestingly, it has also been shown that changes in serum IL-10 expression due to polymorphisms in the IL-10 gene positively correlate with the risks of HCMV infection and disease in transplant recipients [16,17]. IL-10 inhibits the function of antigen presenting cells and the production of proinflammatory cytokines, resulting in impaired CD4 T cell responses [18,19]. This has been suggested to contribute to the immunosuppressive effects seen during acute HCMV infection [8,9]. Many different cell types can produce IL-10, although CD4 T cells seem to be the most important source *in vivo* [19]. HCMV itself contains a gene (*UL111A*) that encodes a viral IL-10 homologue with several similar functions to the cellular protein and that is able to induce cellular IL-10 production from monocytes and dendritic cells (DC) [20–22]. In murine cytomegalovirus (MCMV) infections, IL-10 production from T cells is also important for the establishment of high viral titres [23,24]. Although IL-10 producing T cells specific for both lytic and latent HCMV proteins have been identified in vivo, the mechanisms by which HCMV affects T cell IL-10 secretion during lytic infection are not yet understood in detail [25–27]. It is, however, known that ligation of the lymphocyte phosphatase CD45 can induce T cell IL-10 production [28–30]. The CD45 phosphatase regulates TCR signal strength via its control over the activity of the Src family kinase Lck and, thereby, influences diverse aspects of T cell function [31]. Changes in cytokine production have been linked to CD45 ligation and altered TCR signalling. Both a monoclonal antibody (mAb) directed against the R0 and RB isoforms of CD45 and the lectin galectin-1, which binds to CD45, have been shown to modulate the cytokine secretion profile of treated cells upon CD45 ligation, including by the induction of IL-10 [29,30,32]. As we have previously shown that the extracellular domain of the HCMV glycoprotein pUL11 interacts with CD45 in trans and perturbs T cell signalling and functions, we investigated the effects of pUL11 treatment on IL-10 secretion by primary T cells and considered the underlying changes to T cell signalling [33]. In this study, we show that treatment of PBMC with pUL11 induces IL-10 producing T cells and that supernatants of pUL11 treated cells have anti-inflammatory effects on untreated PBMC. CD45 can act as both a positive and negative regulator of T cell receptor (TCR) signal strength, depending on its expression level, and we show that pUL11 can also have both activating and inhibitory effects on T cell proliferation and on the kinase function of the CD45 substrate Lck.

## Results

### pUL11 induces IL-10 secretion from primary T cells

**Fig 1:**
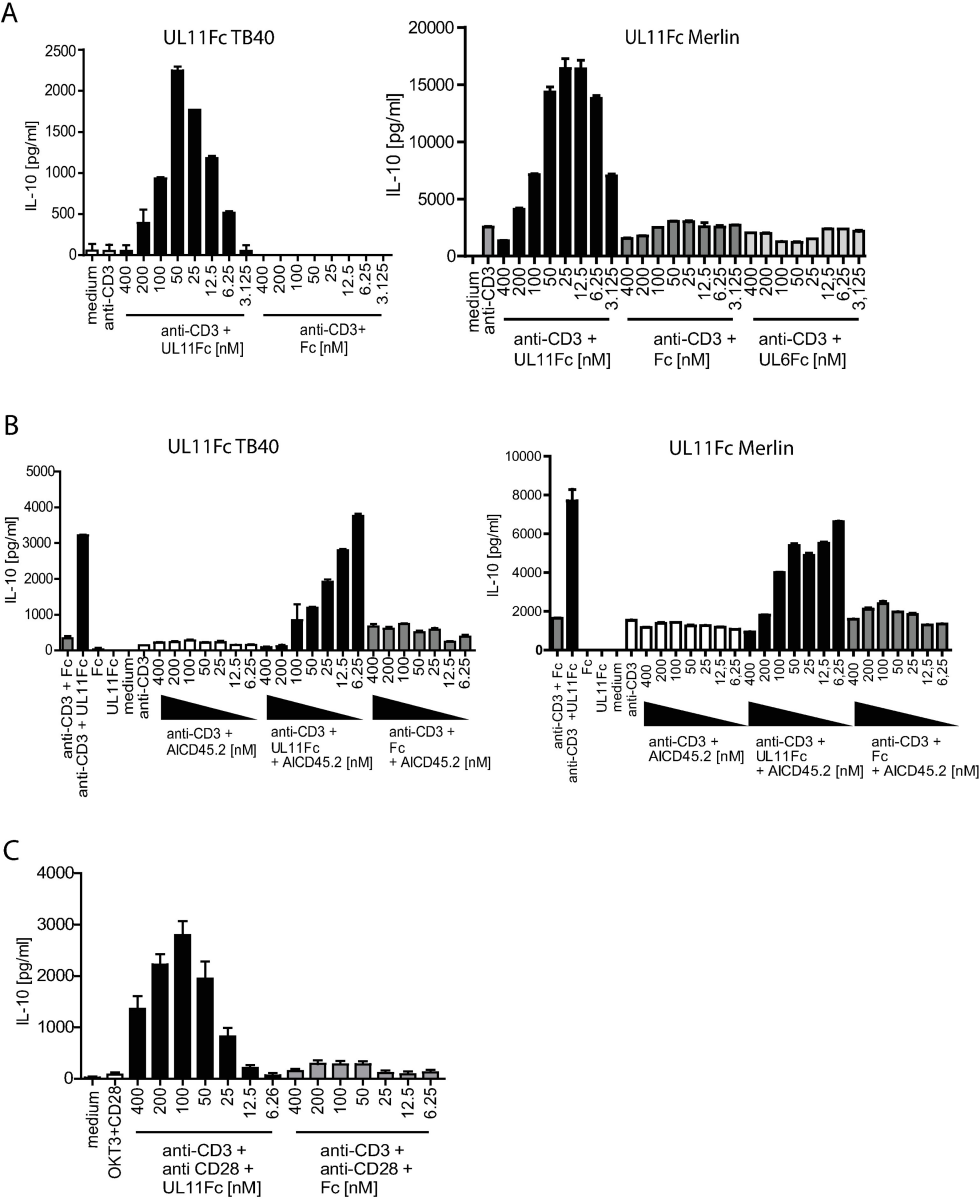
pUL11 treatment leads to the induction of IL-10 secretion. A) PBMC were untreated (medium) or stimulated with anti-CD3 (OKT3) in the presence or absence of UL11Fc (TB40), UL11Fc (Merlin), UL6Fc or the Fc control protein at the indicated concentrations for 48h. B) PBMC were untreated or stimulated with anti-CD3 or treated with 50nM UL11Fc (TB40), UL11Fc (Merlin) or Fc without anti-CD3 (UL11Fc/Fc) or with anti-CD3, following preincubation with the indicated concentrations of anti-CD45 (AICD45.2) where shown, for 96h. C) Enriched primary T cells were untreated or stimulated with anti-CD3 and anti-CD28 in the presence or absence of UL11Fc (TB40) or Fc as indicated, for 48h. IL-10 secretion into the supernatant was measured by ELISA. Single representative experiments from two or three replicates are shown. Error bars show standard deviation of technical replicates.

The HCMV glycoprotein pUL11 interacts with the tyrosine phosphatase CD45, resulting in inhibited TCR signalling and with a range of potential consequences for T cell function [33]. Changes in T cell function resulting from CD45 ligation by other ligands include the induction of IL-10 secretion in cells with activated TCR signalling [29,30,32]. To determine whether pUL11 has a similar effect, we measured IL-10 secretion by pUL11 treated PBMC. The UL11 protein is a transmembrane protein expressed on the cell surface of HCMV infected cells [34,35]. To investigate the effects of pUL11, fusions of the extracellular domain of pUL11 with the Fc domain of human IgG1 (UL11Fc) were used [33] (S1 Fig). This enabled us to investigate the effects of pUL11 in the absence of the complex mixture of other proteins produced during HCMV infection, some of which may also influence cytokine production. The sequence of pUL11 varies between different HCMV strains. To confirm that any effects seen were not limited to the protein from one strain of HCMV, we used fusion proteins of pUL11 derived from both the TB40 and Merlin strains of HCMV. A similar Fc fusion with pUL6, a related protein from the same HCMV gene family, and the Fc domain alone were used as negative controls. PBMC were incubated in the presence or absence of pUL11 or control proteins in combination with the anti-CD3 antibody (OKT3) for two days to induce TCR signalling. Supernatants were harvested and released IL-10 was measured by ELISA (Fig 1A). Increased secretion of IL-10 was observed in the presence of pUL11 from either HCMV strain plus anti-CD3 and was not induced by the control proteins. Titration of pUL11 revealed that IL-10 production did not show a linear correlation with the concentration of pUL11 used; intermediate levels of pUL11 between 12.5nM and 50nM, depending on the strain of virus from which pUL11 was derived, were most effective at inducing IL-10 secretion. The difference in optimum concentration of pUL11 between the viral strains may reflect donor variations or small quantitative differences in the ability of the two proteins to induce IL-10 secretion. The difference in IL-10 secretion level between the pUL11 proteins from the two strains is due to donor variation in IL-10 secretion capacity; neither strain was consistently able to induce higher levels of IL-10 secretion than the other.

To confirm that the effects of pUL11 on IL-10 secretion depended on its interaction with CD45, we used a mAb directed against CD45 (AICD45.2). This antibody had previously been shown to inhibit the interaction of pUL11 with CD45 [33]. In the presence of this antibody, the production of IL-10 was abrogated in a dose-dependent manner by increasing antibody concentrations (Fig 1B). IL-10 secretion was also not induced by pUL11 treatment in the absence of TCR stimulation (Fig 1B). Several different cell types can secrete IL-10 [36]. To determine whether pUL11 treatment induces T cell IL-10 secretion, we enriched T cells from PBMC by negative selection to 98% purity (S2 Fig). After stimulation using plate-bound anti-CD3 and soluble anti-CD28 to induce TCR signalling and co-stimulation, these T cells upregulated IL-10 secretion in the presence of pUL11 (Fig 1C). Titration of pUL11 again resulted in a peak of IL-10 production at intermediate concentrations of pUL11; the curve is slightly shifted with respect to that seen for PBMC, both in terms of peak IL-10 production and of optimal pUL11 concentration, which may reflect differences in the intensity of TCR stimulation between the two experimental settings and variations between donors.

### An HCMV UL11 deletion mutant has a reduced ability to induce IL-10 secretion

**Fig 2:**
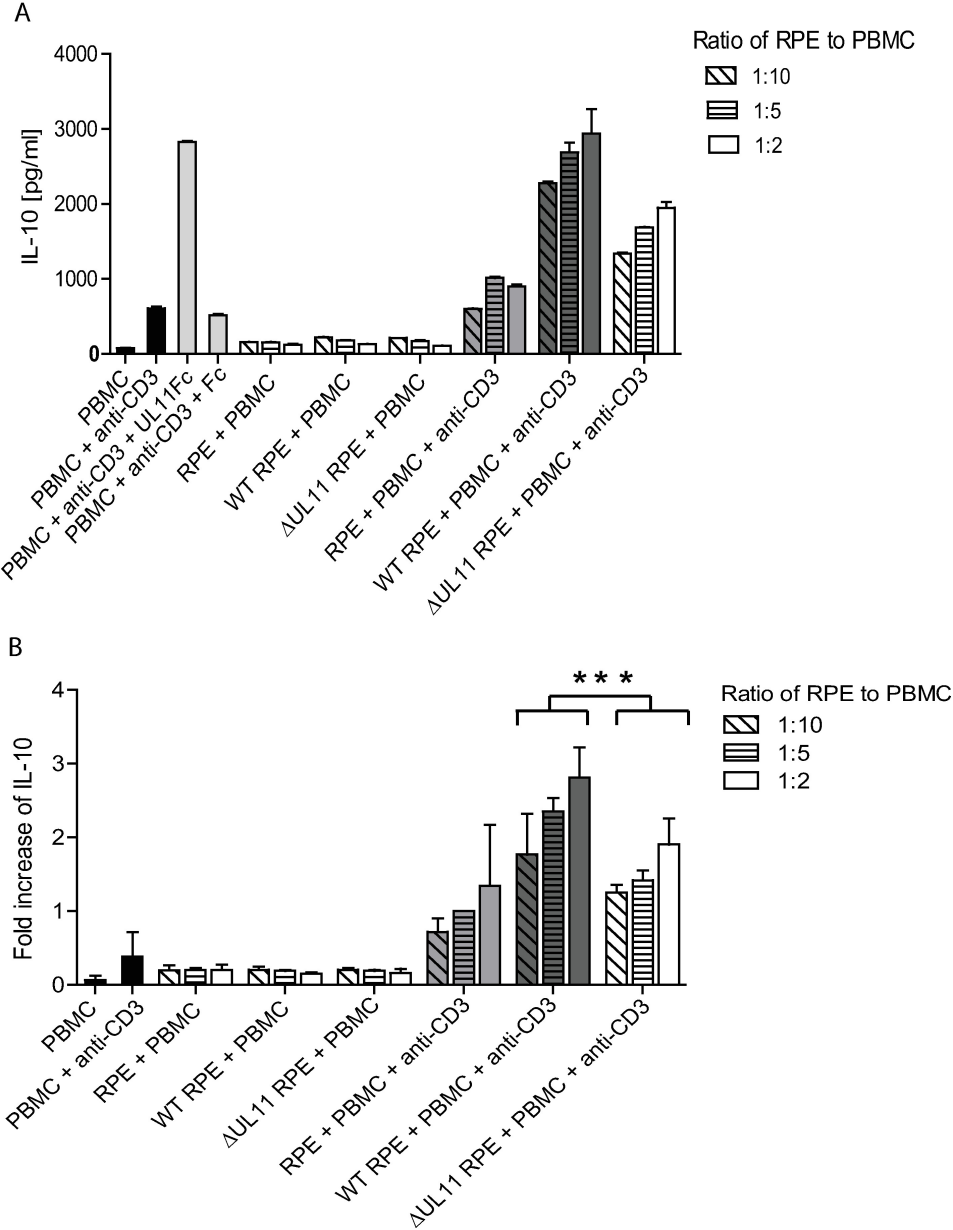
HCMV infected epithelial cells induce IL-10 secretion from PBMC. PBMC were incubated for 48h alone or with 50nM UL11Fc or Fc as a control, or with RPE cells that were uninfected (RPE) or infected with wild type HCMV (WT RPE) or ΔUL11 HCMV (ΔUL11 RPE) at the indicated ratios and in the absence or presence of anti-CD3 (OKT3). The presence of secreted IL-10 was measured by ELISA after 2 days. The experiment was performed three times, using PBMC from different donors. (A) One representative experiment is shown, with IL-10 concentration in pg/ml. Error bars show standard deviation of technical replicates. (B) Mean fold increases in IL-10 concentration over the value for uninfected RPE cells incubated with PBMC at a ratio of 1:5 in the presence of anti-CD3 are shown for the three experiments combined. Error bars show standard deviation. Statistical significance of differences between groups was determined by ANOVA. *p = 0.0005 for the difference in IL-10 expression between supernatants from cultures containing wild type HCMV and those containing ΔUL11 HCMV infected cells.

The induction of IL-10 by the UL11 protein is concentration dependent (Fig 1A). To determine whether the concentration of pUL11 expressed on the surface of HCMV infected cells is suitable for the induction of IL-10 from PBMC in coculture, retinal pigment epithelial cells (RPE) were infected with either the low passage parental Merlin strain of HCMV (referred to here as HCMV wt), or HCMV ΔUL11, from which the *UL11* open reading frame (ORF) has been deleted [35]. Both viruses encode green fluorescent protein (GFP), allowing the approximate determination of infected cell number by flow cytometry. A mouse mAb directed against pUL11 was used to measure the percentage of cells within the culture with pUL11 surface expression by flow cytometry (S3 Fig). After two days of infection, cells from parallel cultures with similar infection rates of HCMV wt and HCMV ΔUL11 (50–80% infected, 17–26% expressing pUL11 over all three experiments, 0%, 2% and 6% differences in HCMV wt and HCMV ΔUL11 infection rates, with the higher infection rate for HCMV ΔUL11) were directly mixed with anti-CD3 treated PBMC at ratios of 1:2, 1:5 and 1:10. After two days of incubation, secreted IL-10 was measured by ELISA. The experiment was repeated three times using PBMC from three different donors (Fig 2). Proximity to HCMV infected cells induced the secretion of IL-10 in anti-CD3 stimulated cells. This secretion was reduced in the absence of pUL11 expression and also required anti-CD3 stimulation. Due to variations in IL-10 production between donors, IL-10 concentrations for each donor were normalized to the level induced by incubation of stimulated PBMC with uninfected RPE cells at a ratio of 1:5. A significant difference in fold increase of IL-10 induction could be seen between the cultures containing HCMV wt and those containing HCMV ΔUL11 infected RPE cells (p = 0.0005). It should be noted that IL-10 secretion was not completely abrogated in the absence of pUL11, indicating the presence of further viral factors capable of inducing IL-10 expression.

### IL-10 production increases over time and is accompanied by a rise in the number of IL-10 producing CD4 T cells

**Fig 3:**
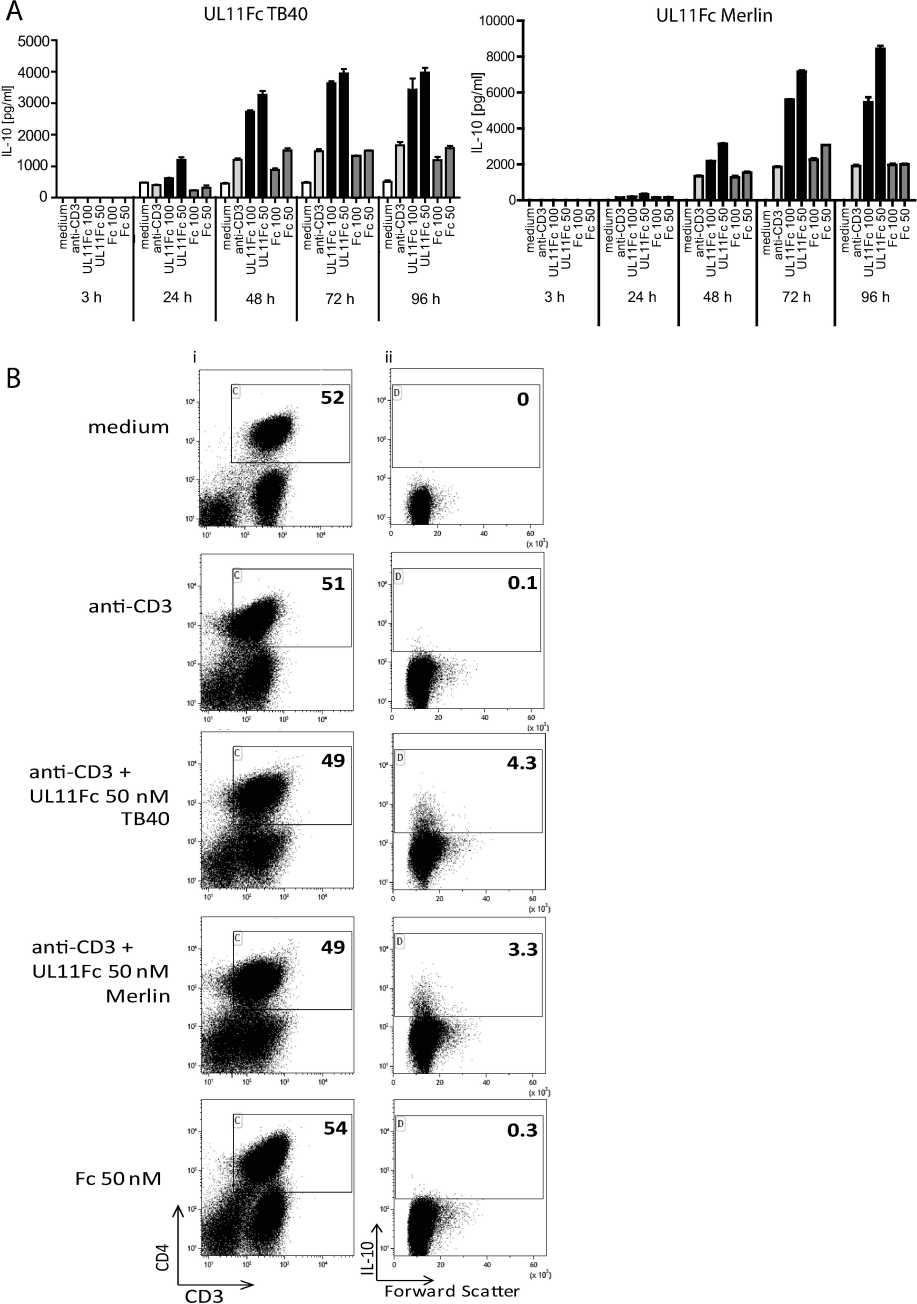
IL-10 secretion following pUL11 treatment accumulates over several days and involves the formation of new IL-10 producing T cells. PBMC were left untreated (medium) or incubated with anti-CD3 (OKT3) or with anti-CD3 and UL11Fc (TB40), UL11Fc (Merlin) or Fc at the (A) indicated concentrations and times or for (B) 72h. (A) Supernatants were harvested and the concentration of IL-10 measured by ELISA after 3 days of incubation. One representative experiment out of two or three is shown. Error bars represent standard deviation of technical replicates. (B) Cells labelled extracellularly with anti-CD3 and anti-CD4, and intracellularly with anti-IL-10 were measured by flow cytometry. Percentages of live CD3^+^ CD4^+^ cells are shown (i) and the IL-10^+^ cells from this compartment (ii). One representative experiment out of five is shown.

The induction of IL-10 by pUL11 treatment could be a result of increased cytokine production by pre-existing IL-10 producing cells, or of the induction/expansion of new IL-10 secreting cells. To distinguish between these two possibilities we first measured the kinetics of IL-10 induction upon pUL11 treatment by ELISA (Fig 3A). The pUL11 induced IL-10 production required 3–4 days to reach its maximum. As the half-life of IL-10 is short (60 minutes [37]), the delay in reaching high IL-10 levels may not be due to the slow accumulation of the cytokine, but rather to a process involving proliferation or conversion of cells to produce IL-10. To investigate the impact of pUL11 on IL-10 producing cells directly, flow cytometry was used (Fig 3B). The proportion of CD4 T cells producing IL-10 increased following three days of treatment with pUL11 from both TB40 and Merlin strains of the virus, more so than following stimulation with the anti-CD3 antibody alone or in the presence of the control protein, indicating that new IL-10 producer cells are induced. Under the conditions used in this experiment, no general expansion of the CD4 T cell compartment was seen; percentages of CD3+CD4+ cells remained between 49% (UL11Fc treated cells) and 54% (Fc control treated cells) (Fig 3B).

### Supernatant from pUL11 treated cells has anti-inflammatory properties

**Fig 4:**
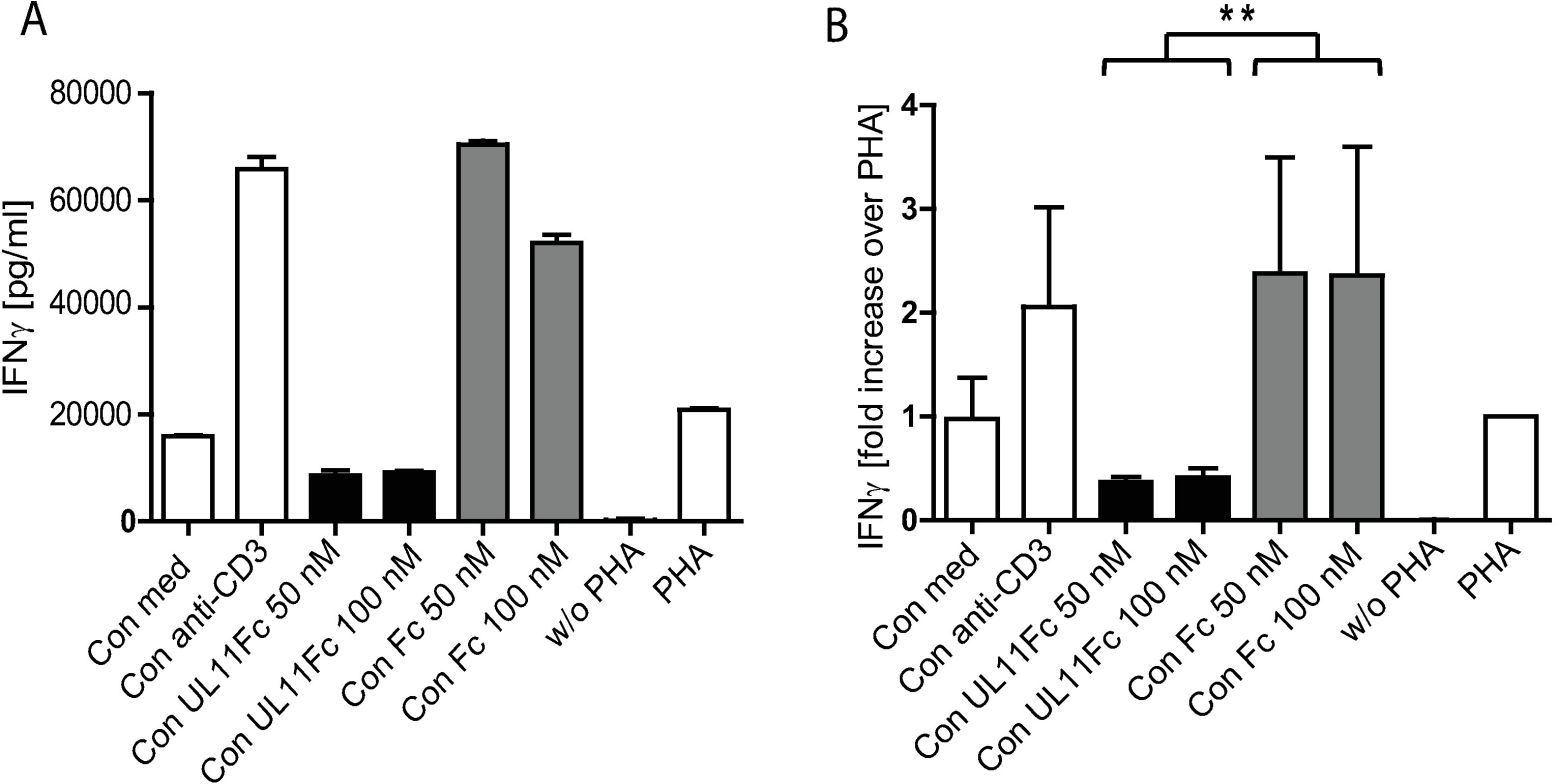
Supernatant from pUL11 treated cells inhibits IFNγ production. PBMC were incubated for 48h in the absence (w/o PHA) or presence of PHA alone (PHA) or together with conditioned medium from untreated PBMC (Con med) or from PBMC treated with anti-CD3 alone (Con anti-CD3) or with the addition of 50nM or 100nM of UL11Fc (Con UL11 50, Con UL11 100) or 50nM or 100nM of the Fc control (Con Fc 50, Con Fc 100). IFNγ concentration in the supernatants was measured by ELISA after 48h of incubation. (A) One representative experiment out of three is shown. Error bars show standard deviation of technical replicates. (B) Fold increase in IFNγ concentration in comparison to that from cells stimulated with PHA alone is shown for the three experiments combined (normalised to values for PHA stimulated cells). Background IFNγ signals present in the conditioned medium were subtracted. Error bars indicate standard deviation. Statistical significance of differences between groups was determined by ANOVA. *p = 0.0034 for the difference between IFNγ expression in supernatants from cultures incubated with conditioned medium from pUL11Fc treated cells and those incubated with conditioned medium from Fc treated cells.

IL-10 is a potent anti-inflammatory cytokine that limits excess immune system activation. Phytohemagglutinin (PHA) is a lectin that induces the production of pro-inflammatory cytokines including IFNγ by PBMC [38]. The action of IL-10 on PBMC includes the inhibition of IFNγ production [36,39]. Treatment of PBMC with pUL11 induces IL-10, but may also affect the secretion of other immunoregulatory cellular factors. For this reason we investigated whether or not conditioned medium from pUL11 treated anti-CD3 stimulated cells has an overall anti-inflammatory effect by measuring the inhibition of PHA-induced IFNγ production by PBMC. While the addition of conditioned medium from control anti-CD3 stimulated cells enhanced the effects of PHA, medium from pUL11 treated cells had a significant inhibitory effect (Fig 4).

### pUL11 treatment of PBMC results in concentration-dependent biphasic effects on TCR driven proliferation

**Fig 5:**
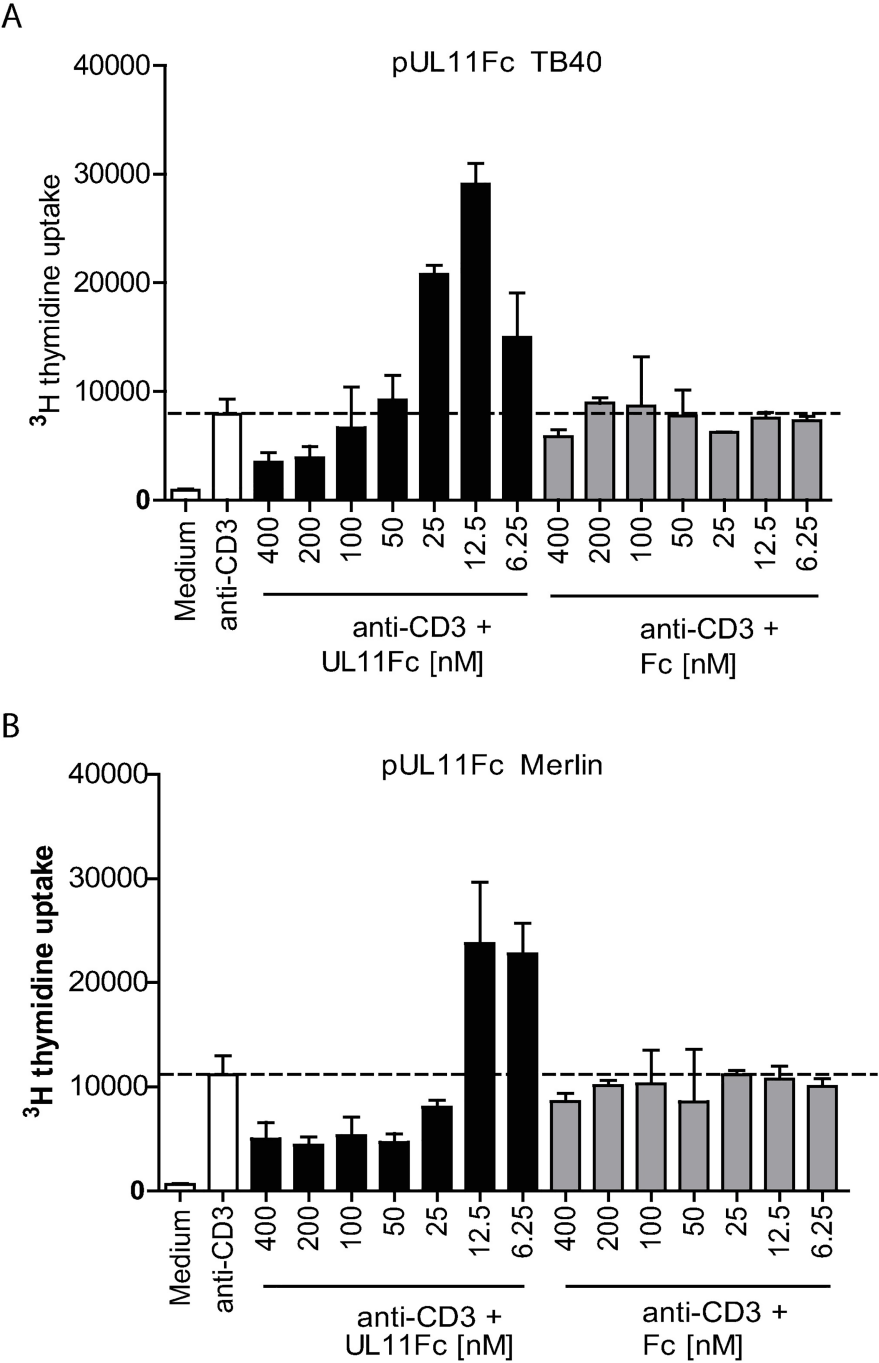
pUL11Fc modulates T cell proliferation in a dose dependent manner. PBMCs were either left unstimulated or stimulated with anti-CD3 (OKT3) together with the indicated concentrations of UL11Fc derived from HCMV strain (A) TB40 or (B) Merlin or with the Fc control protein. Single representative experiments from four (A) or two (B) replicates are shown; error bars show standard deviation of technical replicates.

The main target of the CD45 phosphatase in T cells is the Src kinase p56Lck, which initiates TCR signalling by activating the signal-transducing immunoreceptor tyrosine activation motifs (ITAMs) in subunits of the TCR/CD3 complex [31,40,41]. The kinase activity of Lck is regulated by the phosphorylation of two of its tyrosine residues, one positive and one negative, both of which can be dephosphorylated by CD45. Lck can, therefore, be either activated or inhibited, resulting in hyper- or hyposensitivity to TCR stimulation, depending on the availability of the CD45 phosphatase activity [42]. It has been shown, in mice, that titrating CD45 expression levels has a non-linear effect on T cell proliferation as a result, with reductions in CD45 expression levels down to approximately 30% of wild type resulting in enhanced signalling and proliferation, and larger reductions below this level inhibiting T cell signalling and proliferation [42]. If the interaction of pUL11 with CD45 results in a concentration dependent reduction of the CD45 phosphatase function, titrating the concentration of pUL11 in the experiment would be expected to have similar effects to titrating CD45 expression. Treating PBMC with varying concentrations of pUL11 in the presence of TCR stimulation did result in biphasic effects on proliferation, with lower concentrations of pUL11 enhancing proliferation and higher concentrations having an inhibitory effect, for pUL11 from both the TB40 and the Merlin strains of HCMV (Fig 5A and 5B). Interestingly, at the pUL11 concentrations shown to induce IL-10^+^ CD4 T cells (50nM (Fig 3B)), no increased overall proliferation was seen, again indicating that IL-10 induction is not due to a general T cell expansion.

The non-linear concentration dependent effects of CD45 on T cell proliferation have been shown to be due to its actions on the Lck kinase [42]. Phosphorylation at tyrosine 505 of Lck induces an intermolecular interaction, “closing” and inactivating Lck, and, thereby, preventing proliferation in response to TCR stimulation [43,44]. Phosphorylation of Lck at tyrosine 394, however, is necessary to allow substrates access to the kinase domain of Lck and, in excess, leads to hyperresponsiveness characterized by uncontrolled T cell proliferation [45–47]. Relatively low amounts of active CD45 are sufficient to activate Lck by dephosphorylating tyrosine 505, but even a slight loss of CD45 activity impairs its ability to restrict the kinase activity of Lck by dephosphorylating tyrosine 394, meaning that minor decreases in CD45 activity result in hypersensitive T cell signalling and enhanced proliferation [42].

**Fig 6:**
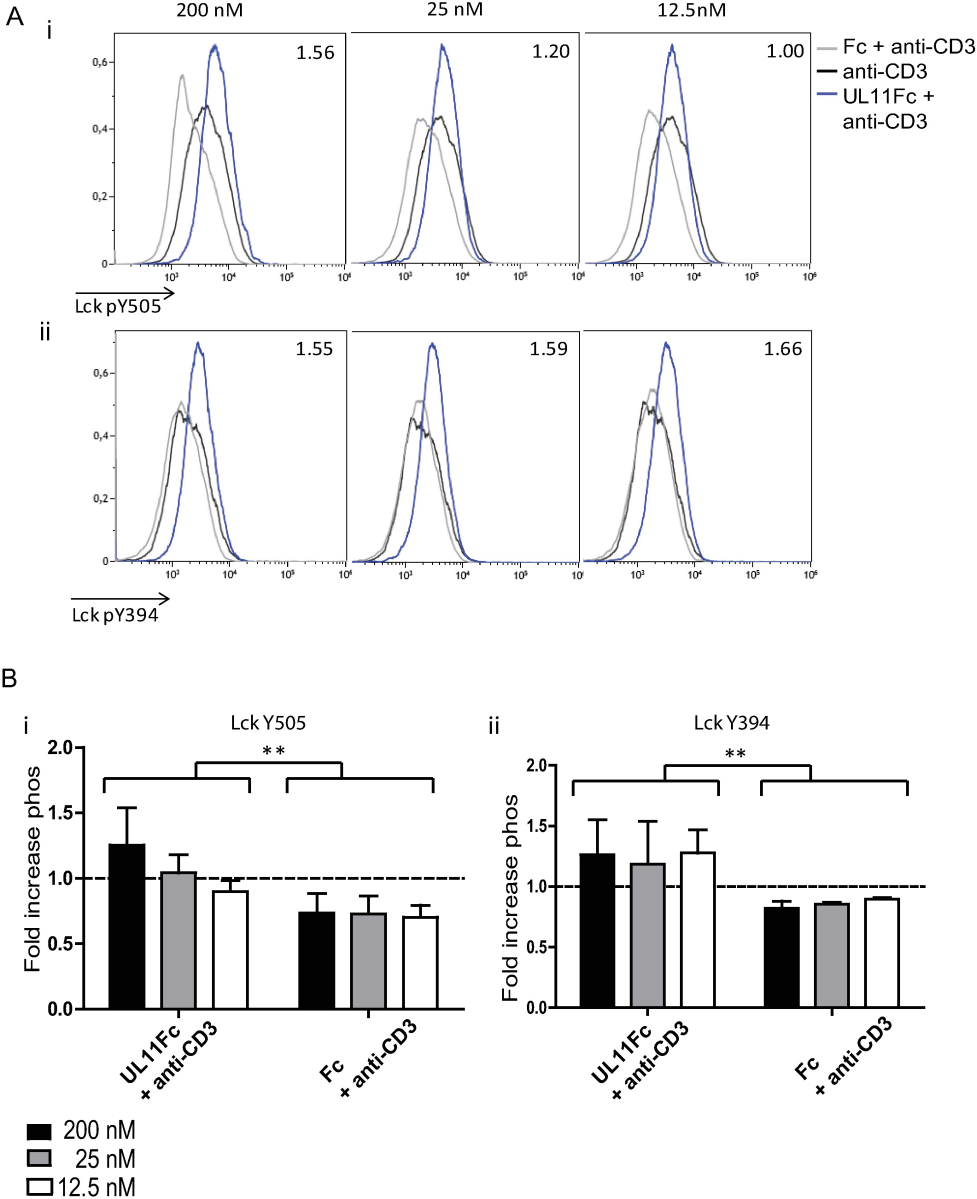
Treatment with pUL11Fc affects phosphorylation of the regulatory tyrosines of Lck. PBMC were incubated with anti-CD3 (OKT3) in the presence or absence of UL11Fc and Fc at the indicated concentrations for three days. Cells were then labelled extracellularly with anti-CD3 and anti-CD4 and intracellularly with anti-Lck pY505 or anti-Lck pY394. The experiment was repeated three times using cells from different donors. Mean fluorescence intensities are shown for Y505 (i) and Y394 (ii) phosphorylation of live CD3+CD4+ cells, fold increases in geometric mean fluorescent intensity normalised to anti-CD3 treated cells are indicated. A) depicts one representative experiment (values for UL11 treated cells). B) shows fold increases in phosphorylation over all three experiments for UL11Fc and Fc treated cells. Error bars indicate standard deviation. Statistical significance of differences between groups was determined by ANOVA. i)*p = 0.0014 for the difference in Y505 phosphorylation between pUL11Fc and Fc treated cells. ii)*p = 0.0043 for the difference in Y394 phosphorylation between pUL11Fc and Fc treated cells.

To explore whether the pUL11-induced proliferation changes in PBMC are linked to a modulation of the available CD45 phosphatase activity and, therefore, the ability of CD45 to dephosphorylate its substrate Lck, we measured changes in the two regulatory phosphotyrosines of Lck. PBMC cultures were treated with differing concentrations of pUL11 for three days and then Lck phosphorylation in CD4 T cells was measured by phosphoflow cytometry (Fig 6A). The experiment was repeated using PBMC from three different donors, with similar results obtained in all three cases. The combined data from all three experiments indicated significant increases in the degree of phosphorylation of both regulatory tyrosines of Lck between pUL11 treated and control treated cells (Fig 6B). Treatment with the control Fc domain resulted in a slight reduction of phosphorylation compared to that seen in cells treated with anti-CD3 alone, possibly due to non-specific steric hindrance of anti-CD3 binding. The UL11 protein affected Lck Y505 and Y394 differently. Whereas only treatment with the highest concentration of pUL11 (200nM) resulted in increased Y505 phosphorylation, increased Y394 phosphorylation could be seen at all concentrations of pUL11 used. These results mirror the effects on Lck phosphorylation of titrating CD45 expression and suggest that pUL11 treatment modulates the available phosphatase activity of CD45 [42]. High concentrations of pUL11 inhibit CD45 phosphatase activity and lead to increased levels of phosphorylated Y505, thereby promoting the adoption of a closed, inactive conformation of Lck. The increased levels of phosphorylated Y394 would result in higher numbers of active Lck kinase molecules, an effect that would predominate at low concentrations of pUL11.

### pUL11 reduces the TCR driven increase in tyrosine phosphorylation of signalling proteins in Jurkat T cells

IL-10 secretion in T cells is known to be influenced by changes in TCR signal strength, such as are induced by alterations in Lck activity [18,40,48]. The induction of IL-10 secretion after treatment with pUL11 requires TCR stimulation, provided here by the anti-CD3 antibody OKT3, and is also highly dependent on the concentration of pUL11 used (Fig 1). Altered signalling states could be generated in the T cells by TCR stimulation in the presence of varying concentrations of pUL11. We therefore examined the effects of pUL11 treatment on the early events of T cell signalling and activation in more detail. As the intensity of signalling effects in primary T cells strongly varies between donors, we used the Jurkat T cell line for these experiments. Although Jurkat T cells differ from primary T cells in their functional responses, the initial signalling events downstream of the TCR in Jurkat cells are similar to those of primary cells, making this cell line a useful model to address this question [49].

**Fig 7:**
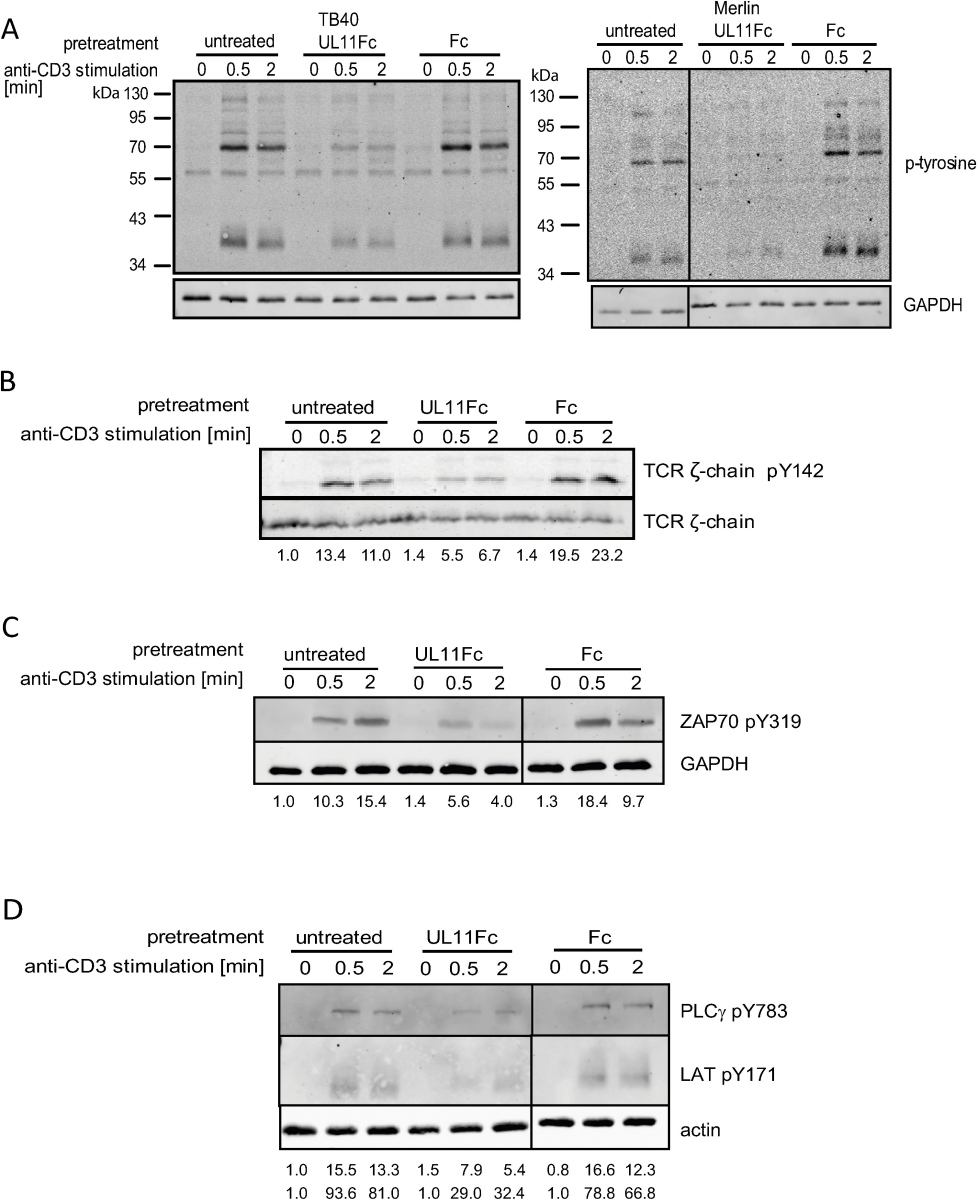
pUL11 reduces the activation of T cells. Jurkat T cells were either untreated or preincubated with 800nM UL11Fc (TB40), UL11Fc (Merlin) and the Fc control protein (A) or UL11Fc (TB40) and the Fc control protein (B, C and D) and then stimulated with an anti-TCR antibody for the indicated times. Activation of TCR signalling was detected by immunoblotting using phospho-specific antibodies; (A) overall tyrosine phosphorylation, (B) TCR ζ-chain Y142 phosphorylation, (C) ZAP-70 Y319 phosphorylation and (D) PLCγ Y738 and LAT Y171 phosphorylation. Quantification of the phospho-specific signals was performed for panels B)-D) and is shown normalised to untreated, unstimulated cells, correlated with loading control intensity.

The treatment of anti-TCR stimulated Jurkat T cells with high concentrations of pUL11 results in a generally decreased tyrosine phosphorylation [50] (Fig 7A). While artificial, this experimental setting allows us to determine whether pUL11 is, in principle, able to influence T cell function by affecting the TCR signalling pathway. In resting cells, elements of the TCR signalling pathway are in dynamic equilibrium, resulting from the combined activities of kinases and phosphatases. Following TCR stimulation, the balance shifts and the ITAMs of the CD3 γ, δ and TCR ζ-subunits are increasingly phosphorylated by activated Lck. The subunit with the largest number of ITAMs is the TCR ζ-chain and this is generally considered the most important for signal transduction [51]. The absence of Lck results in reduced ζ-chain Y142 phosphorylation [52,53]. In pUL11 treated cells TCR stimulation leads to reduced phosphorylation of Y142 of TCR ζ (Fig 7B) in comparison to control cells. This is consistent with a perturbed Lck function in pUL11 treated cells. Following TCR ζ-chain phosphorylation by Lck, the ζ-chain associated protein kinase of 70kDa (ZAP-70) is recruited and phosphorylated, again by Lck [54]. Treatment with pUL11 resulted in reduced phosphorylation of ZAP-70 Y319 upon stimulation (Fig 7C), which is again consistent with reduced Lck activity [53]. ZAP-70 phosphorylation is essential for the subsequent signalling step; the first with a direct link to alterations in T cell function [40].The main substrates of ZAP-70 are the scaffold-forming adaptor proteins Linker for activation of T cells (LAT) and leukocyte phosphoprotein of 76kDa SLP-76 [55]. Which signalling proteins assemble upon LAT is determined by LAT’s phosphorylation state and then influences the degree to which the downstream branches of the signalling pathway are activated, controlling e.g. T cell proliferation and differentiation [41]. Treatment of Jurkat T cells with pUL11 resulted in decreased LAT Y171 phosphorylation (Fig 7D). Phospholipase C (PLC)γ is activated upon interacting with LAT [56]. TCR induced PLCγ Y783 phosphorylation is also reduced by pUL11 treatment (Fig 7D). These results indicate that pUL11 can impair TCR signalling and is likely to be able to induce alterations in T cell function by this route.

## Discussion

The HCMV glycoprotein pUL11 interacts with the lymphocyte surface phosphatase CD45 [33]. CD45 plays important roles in many immune system functions; in the absence of CD45, both humans and mice suffer from a severe combined immunodeficiency (SCID) phenotype. It has been known for many years that signalling through the T cell receptor is completely abrogated in the absence of CD45, B cell receptor signaling is severely disrupted and the functions of other cell types are also affected [31]. It is, however, also clear that CD45 can have far wider effects on T cell function than simply acting as an on/off switch [42]. The level of CD45 expression or activity controls TCR signal strength, which influences a diverse range of T cell features [40].

**Fig 8:**
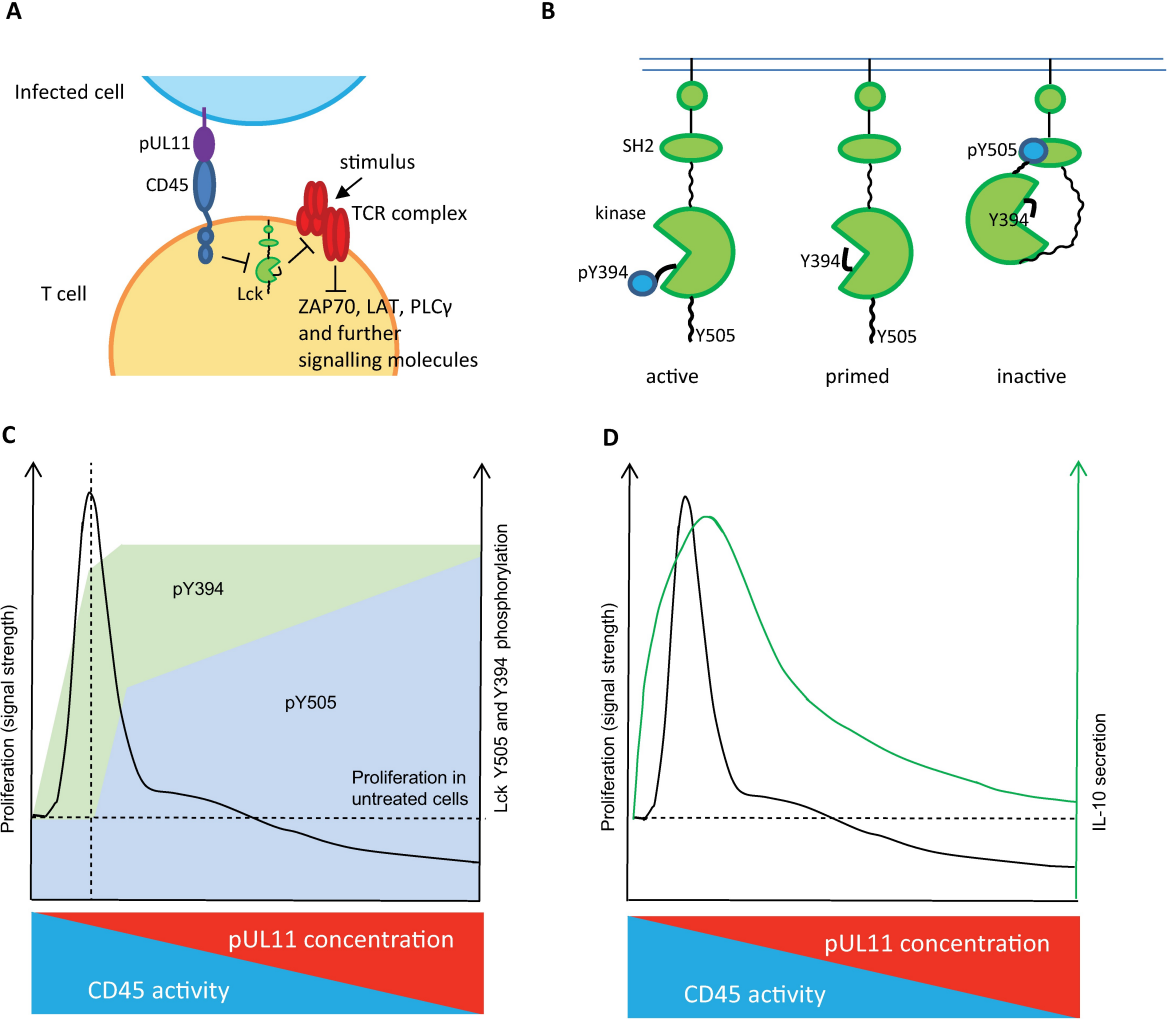
Model. A) pUL11 on the surface of infected cells interacts with T cell CD45. The ability of CD45 to dephosphorylate regulatory tyrosine residues of Lck is reduced. This, in turn, affects the phosphorylation of ITAMs in the TCR complex and other downstream signalling molecules (either positively or negatively, depending on Lck activity). B) Phosphorylation of Lck regulatory tyrosines Y505 and Y394 determines its kinase activity. Y505 phosphorylation induces a closed, inactive state. Y394 phosphorylation is activating, by allowing substrates access to the kinase domain. C) PBMC proliferation in response to stimulation is dependent on the phosphorylation state of Lck Y394 (activating) and Y505 (inhibitory). Peak proliferation (vertical dashed line) is seen when pY394 is increased and diminishes as pY505 increases, inactivating Lck. At the highest levels of pY505, proliferation is inhibited (below the horizontal dashed line). As the concentration of pUL11 is increased, the ability of CD45 to dephosphorylate Lck decreases, shown by increased levels of pY394 and pY505. CD45 preferentially dephosphorylates pY505, hence higher amounts of pUL11 are required to affect Y505 phosphorylation. D) IL-10 secretion induced by pUL11 treatment appears to be influenced by changes in signal strength. T cell IL-10 production is influenced by changes to downstream signalling intermediates such as could be affected by pUL11 treatment.[31,47].

Although no specific cellular CD45 ligand has been identified, lectins that interact with CD45, among other cell surface glycoproteins, have been described. Galectin-1 and Macrophage Galactose-type Lectin (MGL) for example are able to inhibit CD45 activity and to manipulate T cell function [29,57]. Interestingly, the interaction of galectins with CD45 induces changes to T cells that are reminiscent of those seen following treatment with some CD45-specific monoclonal antibodies [28,30]. These mAbs have been shown to reduce rejection of transplants and also to have anti-inflammatory effects [28,58–61]. Both the galectin and mAb CD45 ligands induce a suppressive phenotype in T cells, characterized by changes in cytokine secretion including increased production of the anti-inflammatory cytokine IL-10. We have shown that pUL11 treatment, as has been seen for other CD45 ligands and CD45 antibodies, can affect T cell function (Fig 8) [28–30].

We demonstrated that T cells are a source of IL-10 following pUL11 treatment and that the enhanced IL-10 secretion is both dependent on the interaction of pUL11 with CD45 in the prescence of TCR signaling and associated with an increased proportion of IL-10 producing CD4 T cells. Our results, therefore, support the view that CD45 is a controller of T helper cell decisions, as well as a regulator of activation thresholds and that these properties can be manipulated by the binding of CD45 ligands.

We were also able to demonstrate that pUL11 potentiated induction of IL-10 is part of the repertoire of HCMV infected cells; IL-10 induction was markedly reduced in a deletion mutant lacking pUL11, but not completely abrogated. It is not unusual for HCMV to use more than one mechanism to control important functions; interestingly, HCMV encodes a viral IL-10 homologue, the product of the *UL111A* gene, which diminishes macrophage, dendritic cell and T cell responses *in vitro* and has recently been shown to induce the production of cellular IL-10 in monocytes and DCs, but not T cells [21,22,62–65]. The residual IL-10 induced by ΔUL11 HCMV infected cells may therefore be, at least partly, due to the activity of this protein.

Conditioned medium from cells treated with pUL11 has an anti-inflammatory effect on PHA stimulated PBMC, shown by significant reductions in IFNγ production. IL-10 is a powerful inhibitor of both monocyte dependent and independent T cell IFNγ production making this a likely mechanism for the observed effects of pUL11 [39,66]. IL-10 has a broad range of anti-inflammatory functions; it acts on antigen presenting cells to reduce surface expression of stimulatory and co-stimulatory molecules and inflammatory cytokine secretion and also directly on CD4 T cells [19,36]. T cell proliferation is reduced leading to suppression of Th1 functions in particular. The production of IL-10 is necessary for the host, in order to reduce the immune pathology otherwise associated with infections, but an excess also reduces the effectiveness of immune control [36]. Increased levels of IL-10 have been described in HCMV infected transplant recipients, with larger increases associated with viraemia and disease [8,9,12,15]. The induction of high levels of IL-10 during acute HCMV infections has been speculated to power other immunosuppressive properties of HCMV [8,9]. IL-10 production has also been shown to be important for murine cytomegalovirus (MCMV) infections; by reducing anti-viral responses, establishment of infection is permitted and immune pathology is inhibited [63,67]. IL-10 derived from CD4 T cells in particular suppresses antiviral responses to MCMV, and contributes to the development of high viral titres [23,24]. In the salivary glands, where the virus establishes chronic infection lasting for several months, clearance is ultimately mediated by CD4 T cells. Virus induced CD4 T cell IL-10 production is pivotal to the establishment of this chronic infection, due to its inhibitory effects on CD4 T cell function [68]. This mechanism may have relevance to other mucosal sites of infection, shown to be important for cytomegalovirus transmission [69].

The mechanisms leading to IL-10 secretion have been less well studied in T cells than in monocytes, but appear to be affected by changes in TCR signal strength, such as could result from altered CD45 activity [18,49]. CD45 acts via its substrate the Src family kinase Lck, which plays an essential role in TCR signal transduction by phosphorylating key signalling intermediates. Lck can exist in several phosphorylation and activation states that modulate TCR signal strength [40]. As do all Src family kinases, Lck contains two regulatory tyrosine residues. The inhibitory residue, Y505 in Lck, maintains an intermolecular interaction when phosphorylated, holding Lck in a closed, inactive state [47]. Dephosphorylation of Y505 by CD45 allows Lck to open, forming a “primed” state. Insufficient CD45 activity means that Lck cannot be optimally primed and T cells are less able to transduce incoming TCR signals. CD45 is the only phosphatase that can remove the inhibitory phosphate group from Y505 of Lck and it is for this reason that TCR signalling is completely prevented in the absence of CD45 [70,71]. Titrating CD45 activity does not, however, have a simple linear effect on T cell activation [42]. The second regulatory tyrosine of Lck, Y394, is in a loop adjacent to the kinase domain and must be phosphorylated for the kinase to be functional [47]. Excess Y394 phosphorylation results in increased Lck kinase activity, meaning that T cells are hyperreactive to stimulation via the TCR [45]. The dephosphorylation of these two residues by CD45 are not, however, equally favoured [42]. While small amounts of CD45 (3% of normal levels in mice) can be sufficient to activate Lck by dephosphorylating Y505, close to the full wild type concentration is required to prevent excess Lck activity by dephosphorylating Y394 [42]. A small loss of CD45 activity therefore results in T cells that are hypersensitive to TCR stimulation.

We measured the effects of pUL11 on the phosphorylation of Lck in primary T cells. With the highest concentration of pUL11, the inhibitory Y505 residue showed increased phosphorylation. This increase in Y505 phosphorylation was largely lost at intermediate and low concentrations of pUL11. The activatory Y394 residue, however, showed comparatively increased phosphorylation at all concentrations of pUL11 tested. Similar results have been reported in mice; at CD45 levels of between 10 and 60% of wild type, Y394 phosphorylation was increased 1.4 to 2.1 fold, resulting in enhanced T cell signalling capacity despite some inhibition of Y505 dephosphorylation. At lower CD45 expression levels, the disruption in pY505 dephosphorylation became functionally more important and T cell activation was inhibited [42]. We determined how varying the concentration of pUL11 affects the threshold for TCR signalling using PBMC proliferation as a readout. The results mirrored what is known for CD45 titration; whereas high concentrations of pUL11, which would correspond to low amounts of CD45 activity, inhibited T cell proliferation, when low pUL11 concentrations were used proliferation was markedly increased.

We have previously shown that pUL11 treatment of the Jurkat CD4 T cell line can reduce the rapid induction of tyrosine phosphorylation seen upon stimulation via the TCR [33]. Here we considered the effects of pUL11 treatment on individual proteins immediately downstream of the TCR. The first event following CD3 stimulation, phosphorylation of ITAM motifs in the TCR ζ-chain, was partially inhibited in the presence of pUL11. Phosphorylation of tyrosine 142 of the ζ-chain is exclusively mediated by Lck and its reduction shows the effects of reduced Lck function following pUL11 treatment [52,53]. Disruption in ζ-chain ITAM phosphorylation is likely to reduce the recruitment of ZAP-70, the next protein in the pathway and also an Lck substrate [53,72]. Phosphorylation of ZAP-70 at Y319 was also reduced upon pUL11 treatment. These effects are then passed down the pathway, meaning that phosphorylation of the scaffolding nodes with control over differentiation and cell fate is also affected [55,73]. Tyrosine 171 of LAT is a key residue in the formation of the signalling complex [74,75]. We showed that phosphorylation of this site is inhibited upon pUL11 treatment. PLCγ binding to LAT and its phosphorylation is essential for its activation [76]. Again, the phosphorylation of PLCγ at Y783 was reduced in the presence of pUL11 [56].

We have shown here that the regulation of TCR signal strength and key signalling proteins is affected by pUL11 treatment and it seems plausible that this can underlie the pUL11 induced changes in T cell function. IL-10 induction in T cells, while not yet completely understood, is sensitive to the strength of the input signal and appears to require several pivotal signalling events downstream of the TCR [18,48,77–79]. This is consistent with the observed induction of IL-10 secretion at intermediate pUL11 concentrations corresponding to increased TCR signal strength. The reason that IL-10 induction is not seen when the signal strength is highest is less clear. It is, however, well established that increased TCR signal strength can have inhibitory effects on a range of functions, including cytokine production, via alterations in downstream signalling events [40,80,81].

pUL11 can induce changes in phosphorylation of Lck. It must, however, be borne in mind that although Lck is considered to be the primary substrate of CD45, it is not the only one. pUL11 treatment may also affect other signalling networks with the potential to influence T cell proliferation and IL-10 production. The Src kinase Fyn is also under the control of CD45 and is important in the regulation of TCR signalling strength and its activity [82,83]. CD45 has also been shown to be a negative regulator of Jak/Stat signalling, independently of its effects on Lck [84]. Nevertheless, the TCR signalling pathway appears to be pivotal to the pUL11 induced effects shown here, as in the absence of TCR stimulation no effects of pUL11 treatment were seen.

While pUL11 is able to potentiate IL-10 induction *in vitro*, the situation *in vivo* is likely to be far more complex. The effects of pUL11 appear to be highly concentration dependent; its effects *in vivo* may therefore vary between microenvironments. CD4 T cells are more sensitive to changes in CD45 activity than CD8 T cells are; CD8 T cells from mice expressing only 3% of wild type levels of CD45 retained full cytotoxic function, whereas CD4 T cell helper functions were still impaired in mice expressing 30% of wild type CD45 [42]. pUL11 may therefore be more likely to affect CD4 T cell function *in vivo*. It may also be for this reason that HCMV specific CD8 T cell secretion of IFNγ was not affected by the presence of pUL11 on infected cells [35], in contrast to the results presented here for IL-10 secretion, which is preferentially produced by CD4 T cells [19]. The UL11 protein seems likely to play a role *in vivo*; antibodies to pUL11 have been found in patient sera, implying that the protein is expressed at detectable levels [85]. The protein is variable between strains, but so far no clinical isolates of the virus in which pUL11 is absent or nonfunctional have been confirmed [86]. In a first attempt to investigate whether pUL11 variants from different HCMV strains might differ with regard to their ability to induce IL10 secretion and T-cell proliferation we tested the two pUL11 variants from the TB40 and Merlin strains. However, both pUL11 variants appeared to induce similar effects in our experimental system. The effects of variations in *UL11* sequence on protein function and disease outcome will therefore have to be the subject of future more extensive studies.

The interaction of pUL11 with CD45 is the first example of a viral protein targeting CD45 to induce T cells with anti-inflammatory properties. It is also the first HCMV protein shown to induce IL-10 secretion in T cells. Understanding the mechanisms by which changes in signal strength can influence T cell development and function will contribute to the development of therapies against immune pathologies and may also provide the basis for antiviral treatments.

## Materials and methods

### Ethics statement

Human blood cells were provided by voluntary blood donors in the Institute of Transfusion Medicine, Hannover Medical School. All materials and data were analysed anonymously. The use of human blood cells was approved by the ethics committee of Hannover Medical School.

### Cells and viruses

The Jurkat E6-1 cell line was cultured in RPMI1640 medium supplemented with 10% FCS, 1% penicillin/streptomycin, and 2mM L-glutamine. PBMC were flushed from leukocyte filters used to prepare white blood cell-depleted erythrocytes from healthy voluntary blood donors for transfusion and separated using Biocoll (Merck Millipore, Darmstadt, Germany) density gradient centrifugation. Cells were frozen and stored in liquid nitrogen until usage. PBMCs were cultured in the same medium as Jurkat cells, with the addition of 1mM sodium pyruvate. T cells were isolated from PBMCs using a magnetic negative selection enrichment kit (BD IMag^T^ Human T Lymphocyte Enrichment Set-DM (Becton Dickinson, Heidelberg, Germany)) and used directly after preparation. PBMC (or primary T cells) from at least two different donors were used for each experiment.

RPE cells were cultured in Dulbecco's Modified Eagle Medium: Nutrient Mixture F-12 (DMEM/F12, ThermoFisher Scientific, Darmstadt) supplemented with 10% FCS, 1% penicillin/streptomycin, 2mM L-glutamine.

The two viruses were used in this study were both kindly provided by Martin Messerle: HCMV wt was produced using the bacterial artificial chromosome (BAC)-cloned genome of HCMV Merlin-UL128L^TB40^, which contains a frameshift in *RL13* and, after *UL122*, an IRES followed by the gene encoding GFP [87,88]. HCMV ΔUL11 is based on the same BAC, but with a deletion of the *UL11* ORF and containing the ORF encoding gaussia luciferase in its place (for construction see[35], HCMV HM11DL mutant).

HCMV wt and HCMV ΔUL11 are based on the Merlin strain of HCMV. When propagated in RPE cells, this is a cell associated virus. HCMV wt or HCMV ΔUL11 infected cell cultures were therefore maintained by adding uninfected RPE cells to infected RPE at a ratio of 1:1 or 2:1 after the infected cells reached 100% cytopathic effect as determined by microscopy.

### Protein production

Fc fusion proteins were generated using the predicted extracellular domains of pUL11 from the TB40 and Merlin strains of HCMV and from the predicted extracellular domain of pUL6 from the TB40 strain of HCMV. UL11Fc (TB40), UL11Fc (Merlin) and UL6Fc consist of the predicted extracellular regions of the respective proteins fused to the Fc domain of human IgG1 [33]. The Fc control protein consists only of the Fc domain. All three proteins were expressed from retrovirally transduced 293T cells (UL11Fc (TB40) and Fc control protein) or from adenovirally transduced RPE cells (UL11Fc (Merlin) and UL6Fc) and purified by protein A affinity chromatography as described previously [33,35].

### Statistical analysis

Statistical analysis was performed using GraphPad Prism 5. Two-way ANOVA was used to analyse differences between groups due to the presence of the UL11 protein. p-values are shown as: *<0.05, **<0.01, ***<0.001.

### Jurkat T cell stimulation

2.5 x 10^5^ Jurkat T cells were incubated with 800 nM UL11Fc or the Fc control protein in 100 μl medium for 30 min at 37°C. Cells were stimulated with 100 μl of anti-Jurkat-TCR antibody C305 hybridoma supernatant [89] (kindly provided by B. Schraven, Magdeburg) for the indicated times at 37°C. The reaction was stopped by adding 1 ml ice-cold PBS and cells were immediately centrifuged at 4°C. The cell pellet was lysed with lysis buffer (1% NP-40, 1% N-dodecyl-β-D-maltoside, 50 mM Tris-HCl pH 7.4, 150 mM NaCl, 10 mM EDTA, 2 mM sodium vanadate and protease inhibitor cocktail (Calbiochem, Merck Millipore, Darmstadt, Germany) and incubated on ice for 20 min. The samples were frozen and stored until usage at -20°C. The lysate was centrifuged for 10 min at 12,000 rpm and proteins were separated using SDS-PAGE and transferred to a nitrocellulose membrane. Phosphorylated proteins were detected with phospho-specific antibodies; anti-phospho-tyrosine (4G10) (Merck Millipore, Darmstadt, Germany) anti-TCR ζ-chain p-142 (BD, Heidelberg, Germany), anti-ZAP-70p319 (Merck Millipore, Darmstadt, Germany), anti-LATp-171 (Cell Signaling Technology, Leiden, Netherlands), and anti-PLCγp-783 (Cell Signaling Technology, Leiden, Netherlands). Control antibodies to determine equal loading of the gel were anti-GAPDH (Cell Signaling Technology), anti-TCR ζ-chain (Sigma Aldrich, Taufkirchen, Germany), anti-ZAP-70 (Merck Millipore) and anti-actin (Cell Signaling Technology). Secondary antibodies were IR Dye 800CW Goat anti-mouse IgG, IR Dye 680LT Goat anti-mouse IgG, IR Dye 680LT Goat anti-rabbit IgG and IR Dye 800 CW Goat anti-rabbit IgG (Licor). Imaging and quantification were performed using a Licor Odyssey system with Odyssey Application Software version 3.0.

### PBMC proliferation and cytokine secretion

To measure proliferation of PBMCs in response to pUL11, UL11Fc or Fc control fusion proteins were adsorbed at indicated concentrations together with anti-CD3 (OKT3, 1 μg; purified from hybridoma supernatant) onto Nunc Maxi-Sorb 96-well plates (Sigma Aldrich) for 30 min at 37°C. The plate was washed three times and 1x10^5^ PBMCs per well were incubated in 100 μl of culture medium. After 48 h, 0.4 mCi [3H]-thymidine (Amersham Biosciences, Braunschweig, Germany) was added. 24h later the cells were harvested and incorporated [3H]-thymidine was measured in a beta-counter (Perkin Elmer, Rodgau, Germany).

To measure IL-10 secretion, PBMCs or primary T cells were stimulated with plate-bound anti-CD3 (OKT3, 1μg; purified from hybridoma supernatant) with or without soluble co-stimulatory anti-CD28 (2 μg/ml) together with plate bound UL11Fc or the Fc control protein as in PBMC proliferation experiments. The amount of IL-10 secretion was determined after stimulation by ELISA (IL-10 ELISA MAX Standard set (BioLegend, Fell, Germany)) according to the manufacturer’s instructions. Supernatants were always pooled from triplicate wells of a 96-well plate and used for duplicate ELISA measurements (technical replicates).

### IFN-γ secretion

PBMCs were incubated in Nunc Maxi-Sorb 96 well plates coated with anti-CD3 (OKT3 1 μg; purified from hybridoma supernatant) and UL11Fc or Fc control protein (50 and 100 nM) for four days. Supernatant (conditioned medium) was taken at day four, immediately frozen and stored at -20°C until use.

Freshly isolated PBMCs were incubated together with 50 μl of the conditioned medium as decribed above and stimulated with PHA (1μg/ml) (eBioscience, Frankfurt am Main, Germany) for two days in a total volume of 200 μl. On day two, IFNγ secretion was measured by ELISA (human IFNγ ELISA MAX Standard (BioLegend)) according to the manufacturer’s instructions.

### Virus induced IL-10 secretion

Fresh uninfected RPE cells were added to HCMV wt or HCMV ΔUL11 infected cell cultures as described above. After 48h, to provide an indication of the infection rate of the cells for experiments, GFP expression was measured using flow cytometry. Expression of pUL11 at the cell surface was detected by flow cytometry following staining with a mouse monoclonal antibody specific for pUL11 from the Merlin strain of HCMV [35], kindly provided by Martin Messerle, followed by a PE-coupled goat anti-mouse secondary antibody (Immunotools, Friesoythe, Germany). Infected cultures were used for experiments when the infection rate of the two cultures did not differ by more than 6%, with the HCMV ΔUL11 infected culture having the highest infection rate, and when the infection rate was at least 50%. Sixty-thousand PBMC were mixed with the appropriate number of RPE cells to result in ratios of RPE:PBMC of 1:2, 1:5 and 1:10 and incubated in a anti-CD3 coated 96 well plate. After 2 days, supernatant was harvested and the concentration of IL-10 measured by ELISA as described above.

### Flow cytometry

To determine the phenotypes of T cell subsets after UL11Fc treatment 1x10^5^ PBMCs were stimulated and incubated with UL11Fc or the Fc control protein as above. After 24, 48, or 72 h the cells were labelled to detect surface expression of CD3 and CD4 (using anti-human CD3-APC eFluor 480 Clone SK7 and anti-human CD4-APC Clone SK3, (eBioscience)). Intracellular IL-10 expression was detected (using anti-IL-10-PE, clone JES3-19F1, BioLegend) following 2 h of treatment with Brefeldin A (5μg/ml) (BioLegend) treatment and fixation and permeabilisation using Fixation/Permeabilization concentrate and diluent (eBioscience) according to the manufacturer’s instructions. Live/dead cells were distinguished by exclusion of the Aqua fluorescent reactive dye (ThermoFisher scientific, Darmstadt). Measurements were made on a BD LSRII flow cytometer.

To detect phosphorylated Lck, PBMCs were incubated with anti-CD3 (OKT3) together with UL11Fc or the Fc control protein, as in proliferation experiments, but for three days. Cells were harvested and handled on ice. Live cells were detected by exclusion of dye (Zombie NIR Fixable Viability kit, BioLegend). p-Lck was stained intracellularly and measured with specific antibodies for Lck-p505 (BD Phosflow, anti-human Lck(pY505), BD Heidelberg, Germany) and for Lck-p394 (BD Phosflow. anti-Src (pY418), BD) after fixation and permeabilization of the cells using Fixation buffer and Permeabilization Wash buffer (BioLegend) according to the manufacturer’s recommendation. Measurements were made on a Beckman Coulter FC 500 Analyzer flow cytometer.

### Inhibition of IL-10 secretion

To inhibit IL-10 secretion 1x10^5^ PBMCs were pretreated with the AICD45.2 anti-CD45 antibody (kindly provided by R. Schwinzer) that has been shown to prevent the interaction of UL11 with CD45 [33] for 30 min at 37°C. Pretreated PBMCs were then incubated in 96-well plates coated with anti-CD3 (OKT3 1 mg; purified from hybridoma supernatant) and UL11Fc or Fc control (50 nM) for 2 days. After incubation secreted IL-10 was measured by ELISA.

## Supporting information

S1 Fig Purification of Fc fusion proteins. 1μg of each of the Fc control protein, UL11Fc (TB40) and UL6Fc after SDS-PAGE and Coomassie staining are shown.
(EPS)

S2 Fig T cell enrichment from PBMC. T cells were isolated from PBMCs using a magnetic negative selection enrichment kit (BD IMag Human T Lymphocyte Enrichment Set-DM (Becton Dickinson, Heidelberg, Germany)). Cells were labelled with markers for T cells (CD3), B cells (CD19) and NK cells (CD56) and the presence of these markers determined by flow cytometry in the initial PBMC culture (left panels) and after T cell enrichment (right panels). Percentages of live cells positive for each marker are shown.
(EPS)

S3 Fig Measurement of pUL11 expression in HCMV infected epithelial cells. Retinal pigment epithelial cells (RPE) were infected with either the parental Merlin strain of HCMV (HCMV wt), or HCMV ΔUL11, from which the UL11 ORF has been deleted. Both viruses contain the gene encoding green fluorescent protein (GFP). Cells were labelled using a mouse monoclonal antibody directed against pUL11 or an isotype control antibody, detected with a PE-coupled goat anti-mouse antibody. Percentages of live GFP positive (infected) and PE positive (expressing pUL11) cells are shown.
(EPS)
